# A Morphometric Screen Identifies Specific Roles for Microtubule-Regulating Genes in Neuronal Development of P19 Stem Cells

**DOI:** 10.1371/journal.pone.0079796

**Published:** 2013-11-18

**Authors:** Julia Arens, Thanh-Thuy Duong, Leif Dehmelt

**Affiliations:** Department of Systemic Cell Biology, Max Planck Institute of Molekular Physiology, and Fachbereich Chemische Biologie, Dortmund University of Technology, Dortmund, Germany; Stanford University School of Medicine, United States of America

## Abstract

The first morphological change after neuronal differentiation is the microtubule-dependent initiation of thin cell protrusions called neurites. Here we performed a siRNA-based morphometric screen in P19 stem cells to evaluate the role of 408 microtubule-regulating genes during this early neuromorphogenesis step. This screen uncovered several novel regulatory factors, including specific complex subunits of the microtubule motor dynein involved in neurite initiation and a novel role for the microtubule end-binding protein EB2 in attenuation of neurite outgrowth. Epistasis analysis suggests that competition between EB1 and EB2 regulates neurite length, which links its expression to neurite outgrowth. We propose a model that explains how microtubule regulators can mediate cellular morphogenesis during the early steps of neuronal development by controlling microtubule stabilization and organizing dynein-generated forces.

## Introduction

Neurons grow specialized cell protrusions that form the basis of highly interconnected cellular networks in the brain [1]. The formation of these protrusions is regulated by intracellular factors, which are themselves controlled by the extracellular environment and the cellular differentiation state. The cytoskeleton, in particular filamentous actin and microtubules, play a key role in this process by altering the mechanical properties of the cell [2]. Actin can form distinct, dynamic supramolecular structures that play multiple roles in cellular morphogenesis. On the one hand, anti-parallel filament bundles, such as stress fibers or arcs can drive cell contraction, and on the other hand, parallel or branched actin filament assemblies such as in filopodia or lamellipodia, can drive cell protrusion. In contrast, the role of microtubules in cellular morphogenesis is less well characterized. It is well accepted that the shape of mitotic spindles emerges from direct interplay between dynamic microtubules and associated motors, however, apart from this well-studied example, microtubules are otherwise mostly seen as tracks for directional transport of cellular cargos. Only more recently, instructive roles for microtubules to control cellular structure and function were proposed [3]. In previous studies, we found that the microtubule motor cytoplasmic dynein can power cellular shape changes in the absence of actin dynamics [4], suggesting that microtubules might play an active role in morphogenic processes. Here, we extended on these observations and performed a morphometric screen in P19 stem cells to analyze the role of microtubule-regulating genes in early neuronal development. Using this strategy, we identified several regulators, which influence neurite formation.

## Results

### Quantification of siRNA induced gene knockdown phenotypes

**Figure 1:**
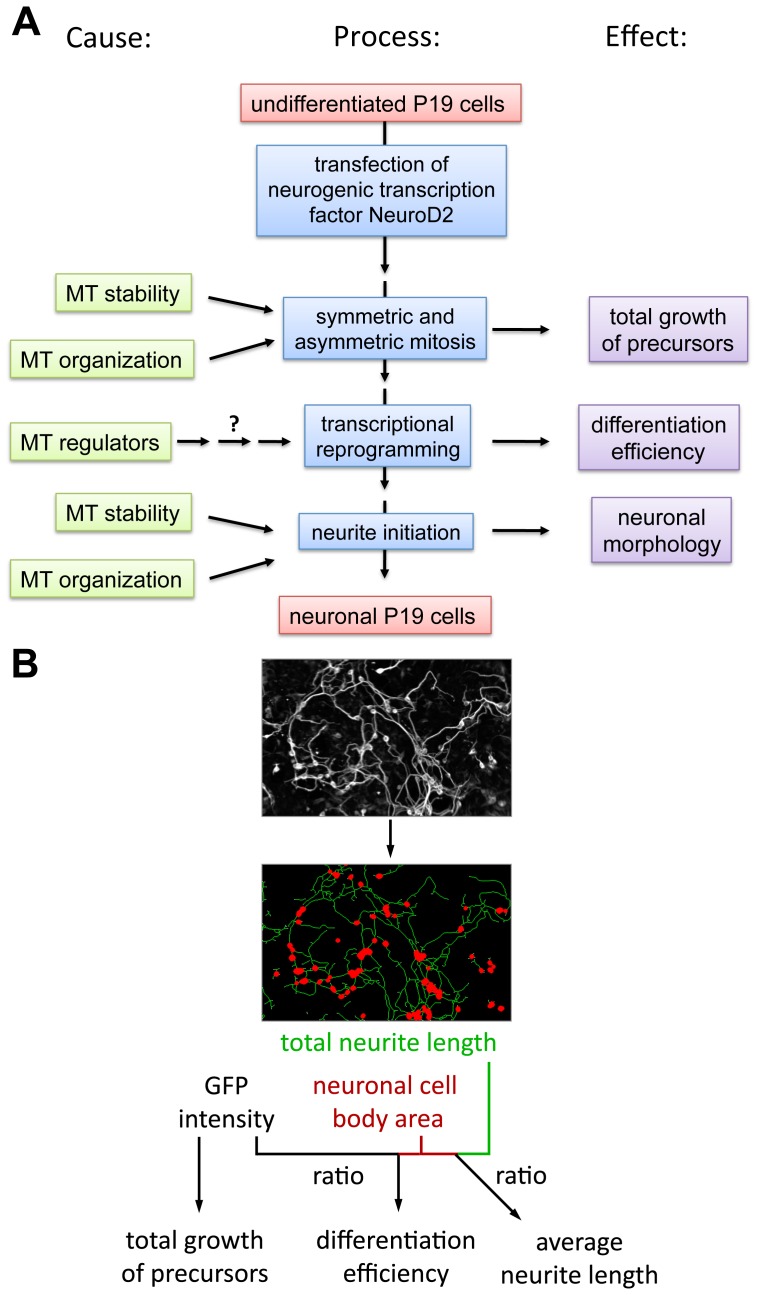
High-content screen for analysis of microtubule-regulating genes during neuronal development of P19 stem cells. A: Microtubule-dependent processes and consequence of their disruption for neuronal development. B: Concept for determination of precursor growth, differentiation efficiency and average neurite length via image processing and image quantification.

To study the role of microtubule-regulating genes in neuronal development, an automated morphometric screen was performed in P19 stem cells by combining the induction of neuronal differentiation with efficient gene knockdown via co-transfection of a neurogenic transcription factor and siRNA oligonucleotides [5] (Figure 1A). Our library of siRNA oligonucleotides covers 408 candidate genes, including microtubule-associated proteins, motor protein subunits, tubulin isoforms and tubulin modifying enzymes. In the primary screen, cytosolic EGFP, the neurogenic transcription factor NeuroD2 and a mixture of 4 independent siRNAs targeting individual microtubule regulators were co-transfected in 384-well plates. Then, secondary screens were performed to test if knockdown phenotypes were consistent using individual siRNAs (see Materials and Methods for details). Transfection of *NeuroD2* leads to neuronal differentiation [6], accompanied by loss of the stem cell marker OCT4 and high-level expression of neuronal markers (Figure S1). Protein knockdown of known neuronal genes, such as *Tubb3* (β-III-tubulin) or *Mtap2* (microtubule associated protein 2) was highly effective and selective under these conditions (Figure S2). However, while the application of siRNA oligo mixtures increases the chances of protein knockdown, it might not always be complete. Thus, the absence of an observed phenotype could also be due to inefficient protein knockdown. To determine the effect of both weak and strong gene suppression with high sensitivity, triplicates of 4-point titrations of siRNA oligonucleotide concentration were prepared. After 4 days in culture, nuclei were stained using Hoechst 33258 and neuronal β-III tubulin via immunocytochemistry. From each well, images of 6 microscopic fields were obtained, which covered a total area of 3.2 mm^2^ containing approximately 1000 neurons.

**Figure 2:**
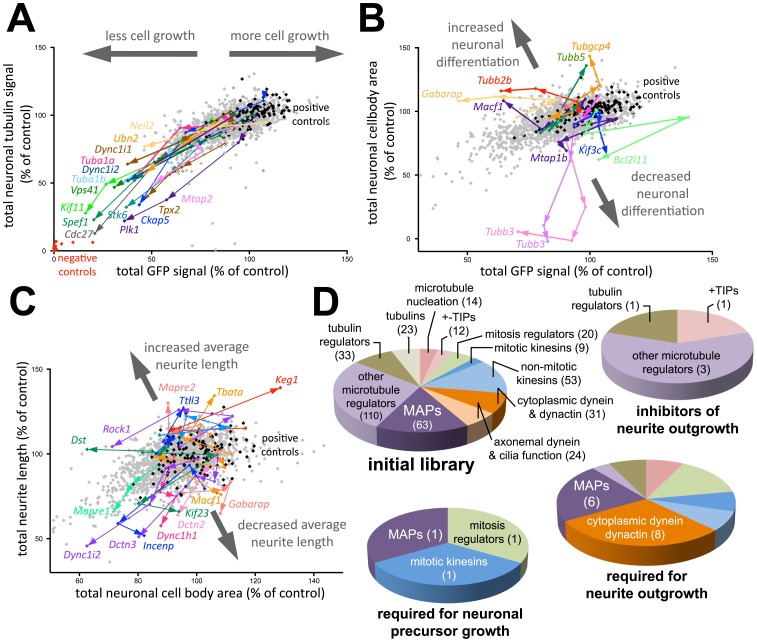
Classification of phenotypes induced by knockdown of microtubule-regulating genes. Positive controls (differentiated but no siRNA) and negative controls (not differentiated) are indicated by black and red spots, respectively. Titrations of siRNA (0.5–4 pmol/well) targeting microtubule-regulating genes are indicated by arrows pointing toward higher concentrations. Each data point is an average of 3 independent screen repetitions. Grey spots correspond to candidates, which did not induce a strong phenotype. A: Determination of neuronal precursor cell growth by measuring changes in the total EGFP signal. B: Determination of neuronal differentiation by comparing the ratio between the EGFP and total neuronal cell body area relative to positive controls. Thick grey arrows indicate modulation of neuronal differentiation efficiency. C: Measurement of average neurite length by comparing the ratio between total neurite length and neuronal cell body area relative to positive controls. Thick grey arrows indicate modulation of average neurite outgrowth. D: Functional classification of microtubule-regulating genes in the initial library and in three phenotypic hit categories based on stringent selection criteria. Numbers represent the number of genes in each category.

To determine the effect of siRNA treatment on neuronal development, quantitative morphometric image analysis was performed using NeuriteQuant [7] (Figure 1B). Our analysis focused on the following parameters: a) cell growth to determine proliferation of neuronal precursors, b) neuronal markers to evaluate differentiation of precursors to post-mitotic neurons, and c) neurite length to investigate neurite outgrowth. Figure 2 shows average intensity and morphological measurements of all screen repetitions that were used to derive these parameters. Each graph shows the relative contribution of two morphometric parameters on the x- and y-axes. Titrations of increasing siRNA concentration are represented by colored arrows.

Some siRNAs induced a correlated reduction of total EGFP fluorescence intensity and neuronal β-III tubulin signal (Figure 2A). This represents a reduction in the initial growth of proliferating EGFP-transfected precursors, which consequently also leads to a proportional decrease in the number of differentiated neurons and therefore also to a reduction in total neuronal β-III tubulin. Genes related to mitosis are overrepresented in this phenotypic class (66% vs 7.4% of the candidate genes in the initial library; Figure 2D). Table S1 lists all gene targets, which affect proliferation of precursors and shows the average reduction of the total EGFP-fluorescence per field compared to positive controls in units of standard deviations. In addition to several central cell cycle regulators, genes, which have important household functions (for example the HOPS-complex subunit *Vps41*) also affect this phenotype. This is in agreement with their essential roles in cell proliferation. Unexpectedly, the screen also identified *Spef1* as a gene that plays a role in precursor proliferation. *Spef1* is an evolutionary conserved microtubule bundling protein associated with cilia or flagella that is expressed predominantly in testis, but also found in EST libraries from embryos or brain [8].

To determine neuronal differentiation, the total cell body area of β-III tubulin positive cells was measured, which is proportional to the number of neurons per field. In order to extract a measurement of neuronal differentiation that is independent of precursor proliferation, the ratio between the total neuronal cell body area and the total EGFP signal was measured in comparison to neurons that were not treated with siRNA (Figure 2B, grey arrows indicate increased or decreased neuronal differentiation, see also Tables S2 and S3). Only few genes induced marginal changes in neuronal differentiation. This was not surprising, as the mechanisms that control neuronal differentiation are primarily based on gene transcription, which is currently not thought to involve microtubules. Knockdown of our neuronal cell body indicator β-III tubulin (*Tubb3*) led to an artificial, apparent reduction of neuronal differentiation, as differentiated neurons were not detected in this condition.

A distinct set of siRNAs induced a change in the proportion of total neuronal cell body area and total neurite length of differentiated P19 cells, suggesting a specific role of gene products in neurite initiation and/or subsequent neurite elongation (Figure 2C). This effect was quantified similarly to neuronal differentiation efficiency by analyzing the ratio between total neurite length and total neuronal cell body area (grey arrows in Figure 2C, Tables S4 and S5). Genes that code for subunits of cytoplasmic dynein/dynactin microtubule motors are overrepresented in the set of candidate regulators that are required for neurite outgrowth (29.6% vs 7.9%; Figure 2D). Furthermore, we found that in addition to the motor containing dynein heavy chain, a specific set of dynein/dynactin subunits plays a role in neurite formation. This suggests that those subunits form a complex together with the heavy chain to regulate neurite formation.

Knockdown of a small set of candidates lead to increased neurite length, suggesting that those candidates act as inhibitors of neurite outgrowth. For example, knockdown of the Rho kinase isoform *Rock1* lead to this phenotype. This observation is consistent with the proposed function of Rho kinase as a gate to prevent axon initiation and elongation [9]. Interestingly, this screen also identified novel inhibitors of neurite outgrowth, including the cytoskeletal crosslinker dystonin (*Dst*), the transcriptional regulator spatial (*Tbata*), and the end-binding protein EB2 (*Mapre2*). The identification of EB2 as a negative regulator of neurite outgrowth was surprising due to the function of the EB1/EB2/EB3 protein family as microtubule stabilizers. We therefore analyzed this gene family in more detail below.

### Competition between end-binding proteins in early neuromorphogenesis

**Figure 3:**
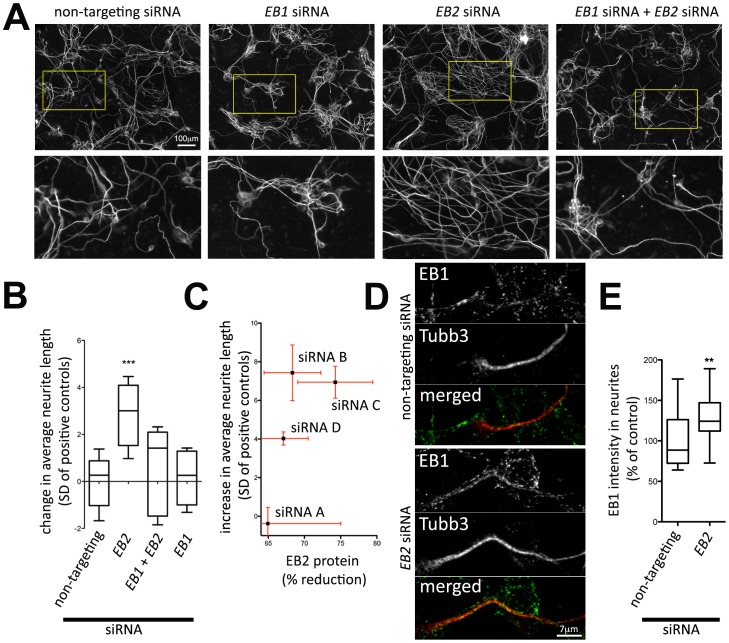
Epistasis analysis suggests competition between EB1 and EB2 in regulation of neurite outgrowth. A: P19 cells co-transfected with neurogenic transcription factor NeuroD2 and 2pmol siRNA oligonucleotides for each target gene were stained with antibodies against neuronal β-III-tubulin (Tubb3). The total amount of siRNA oligonucleotides per well in a 384-well plate was kept constant at 4pmol (non-targeting siRNA was added, if necessary). B: Quantification of neurite outgrowth modulation analogous to Figure 2C. (*: p<0.05; ***: p<0.001; one-way ANOVA with Dunnett’s post-test using non-targeting siRNA as the control group, n = 6). C: Plot of the reduction of EB2 protein levels measured via western blot vs the increase of neurite outgrowth for different siRNAs targeting EB2. Error bars represent the standard error of the mean (n = 3). Additional western blot analyses are shown in Figure S3. D: Confocal z-projections of partially extracted, methanol fixed neuronal differentiated P19 cells stained with antibodies against neuronal β-III-tubulin (Tubb3) and EB1. E: Quantification of average EB1 signals within neurites of P19 cells. Confocal z-projections of EB1 signals were masked based on the neuronal β-III-tubulin signal and average intensities were calculated within those masked regions (**: p<0.01; Student’s t-test, data obtained from 4 independent knock-down experiments).

The dynamic growth and shrinkage of microtubules is thought to be essential for many morphogenetic processes such as the formation of the mitotic spindle. However, for the formation of neurites, relatively stable and long-lived microtubules are required [10]. The microtubule plus-end binding protein EB1 is thought to be a key regulator of microtubule dynamics as it forms a hub for interactions with several additional microtubule regulators and is thought to act as an integrator of these activities [11]. The related family members EB2 and EB3 are similar in structure but less well characterized. Surprisingly, we found that knockdown of EB2 led to a significant increase in average neurite length, suggesting that it is a negative regulator of neurite outgrowth. This was surprising, as members of this family are generally thought to stabilize microtubules [12], [13], and therefore are expected to stimulate neurite outgrowth. This effect of EB2 knockdown was well reproduced in the secondary screens (Table S6). Here, a positive correlation (Pearson’s r = 0.74) between protein knockdown and neurite outgrowth was observed (Figure 3C).

To analyze, if a genetic interaction between EB proteins is causal for this phenotype, genetic epistasis experiments were performed via combined knockdown of EB family members (Figure 3). Knockdown of EB1 alone had no effect, however, knockdown of EB1 abolished EB2 induced outgrowth (Figure 3A and B). This suggests that there is a competition between EB1 and EB2 for regulating neurite outgrowth. Such a competition is consistent with recent studies using non-neuronal cells, in which the three end-binding proteins EB1/EB2/EB3 differ in their stabilizing effect on microtubules and compete for the same binding sites at growing microtubule plus tips [13]. In another recent study, EB2 knock-down resulted in an increase of EB1 signal along the microtubule lattice, suggesting that EB1 and EB2 can also compete for binding sites along the full length of microtubules [14]. Of the three proteins, EB2 was shown to be the weakest stabilizer. This difference in microtubule stabilization is thought to be due to the much weaker affinity of EB2 to the microtubule stabilizing plus-tips CLIP-115 and CLIP-170 compared to EB1 [15]. Therefore, EB2 knockdown might unmask binding sites for EB1, which in turn recruits those additional stabilizers, leading to enhanced microtubule and neurite growth. Indeed, we found that EB2 knock-down lead to increased EB1 signal within neurites in methanol-fixed P19 cells (Figure 3D and Figure 3E). In this fixation procedure, the EB1-microtubule interaction is well preserved due to the quick fixation procedure and the cytosolic fraction of EB1 is partially extracted due to permeabilization of the plasma membrane. Microtubules are very dense within neurites and signals from individual plus-tips overlapped and therefore made it impossible to analyze them individually. Thus, the observed increase of EB1 signal after EB2 knock-down could either be due to an increase in EB1 at microtubule tips or due to an increase in EB1 along the microtubule lattice. Westernblot analysis (Figure S3) and immunostaining (Figure S4) of cells that were fixed with an intact, non-permeabilized plasma membrane revealed no or very little effect of EB2 knockdown on EB1 signals, suggesting that potential compensation on the level of protein expression only plays a minor role.

### The role of cytoplasmic dynein/dynactin in neurite formation

**Figure 4:**
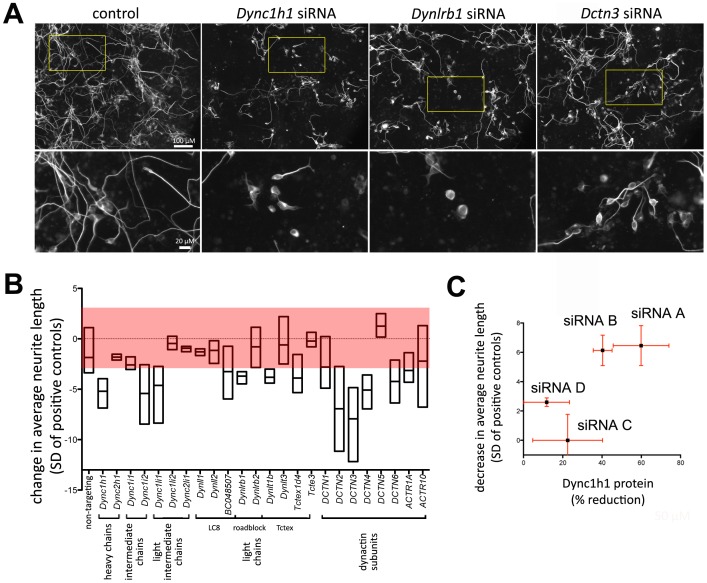
Dynein/Dynactin modulates neurite formation. A: Representative microscopic images of P19 cells acquired in the primary screen, transfected with neurogenic transcription factor NeuroD2 and 4pmol of siRNA oligonucleotide mixtures targeting Dynein/Dynactin subunits. B: Effect of dynein/dynactin subunit knockdown on average neurite length. The boxed area indicates the ±3 standard deviation threshold for potential candidate identification in the primary screen. C: Plot of the decrease in average neurite length vs the reduction of Dync1h1 protein levels measured via western blot for different siRNAs targeting *Dync1h1*. Error bars represent the standard error of the mean (n = 3).

The minus-end microtubule motor cytoplasmic dynein plays important roles in transport of intracellular cargos and in the organization of microtubules. For example, this motor plays important roles during the formation of the mitotic spindle or for specifying the cell center. Specificity with regard to these diverse processes is conveyed via the subunit composition of dynein complexes [16]. In order to relate dynein composition to neurite outgrowth, we focused our analysis on all known subunits of cytoplasmic dynein (Figure 4). Combined western blot and morphometric analysis showed a positive correlation (Pearson’s r = 0.79) between dynein heavy chain 1 (*Dync1h1*) levels and neurite outgrowth (Figure 4C and Figure S4). This demonstrates, that neurite outgrowth is dependent on *Dync1h1*. Despite strong neuronal tubulin staining, the majority of *Dync1h1* suppressed neurons formed short or no neurites (Figure 4A), suggesting that the initial formation of neurites was blocked. This is consistent with a function of a cortical population of cytoplasmic dynein, which can push microtubules to focus an outward force at the plasma membrane, leading to the initiation of neurite-like protrusions[4]. Interestingly, only a subset of dynein subunits is required for neurite outgrowth (Figure 4A, B and Table S4). For example, knockdown of the heavy chain isoform 2 of cytoplasmic dynein (*Dync2h1*) or the light intermediate chain *Dync2li1* did not affect neurite outgrowth. This is consistent with the role of a complex between this heavy chain and this light intermediate chain in driving retrograde intraflagellar transport [17], [18]. Complexes containing the heavy chain isoform *Dync1h1* and the remaining subunits are responsible for all remaining processes mediated by cytoplasmic dyneins. Intermediate chains link the heavy chains to light intermediate and light chains, as well as to the associated dynactin complex. We find that only one of the two intermediate chain isoforms, *Dync1i2*, is required for neurite outgrowth. In previous studies, distinct intermediate chain isoforms were found to convey cargo specificity for vesicular transport [19]. Thus, *Dync1i2* might function together with its associated light intermediate and light chains to define dynein complex specificity for neurite outgrowth.

We also found that all dynactin subunits except for *Dctn5* (Dynactin p25) reduced average neurite outgrowth (Figure 4B). In previous studies of *Neurospora* dynactin subunits, the null mutant of that specific subunit was uniquely deficient in dynein-dependent nuclear movements, while other dynactin subunit mutants showed more general defects [20]. Similarly, in COS7 cells, knockdown of *Dctn5* only affected early and recycling endosome movements but not other dynein dependent processes, such as late endosome movements or mitotic spindle formation [21]. In those cells, *Dctn5* interacts and cooperates with *Dctn6* (Dynactin p27) to form a peripheral “pointed-end” complex within dynactin to perform those functions. However, in our analyses, *Dctn6* and *Dctn5* differentially affected neurite outgrowth. Thus, *Dctn6* might play a distinct role in neurite formation that is independent of *Dctn5*. Taken together, our data suggest that the *Dctn5* subunit might contribute to functional specificity in a subset of dynein mediated processes and is not essential for dynein function in neurite outgrowth.

## Discussion

In this study, we present a comprehensive morphometric siRNA-based screen to study the role of all known microtubule-regulating genes in early neuronal development. As a model system, we chose P19 cells, which express the stem-cell marker Oct4 only in the undifferentiated stage and several pan-neuronal markers, such as β-III-tubulin and MAP2 only in the neuronal differentiated stage. In contrast to embryonic stem (ES) cells, which have properties similar to the inner cell mass, undifferentiated P19 cells are derived from embryonic carcinoma and thought to correspond to a more advanced developmental stage, the primitive ectoderm [22], [23]. Presumably due to this advanced stage, they can differentiate into neurons more rapidly. Compared to primary neurons, stem cells still undergo mitosis before their differentiation, which makes it difficult to study the role of mitotic microtubule regulators in neuronal development. We therefore excluded microtubule regulators that interfered with neuronal precursor growth from our analysis. On the other hand, the initial neurites formed by neurons *in vivo* are typically lost during their isolation, and the regrowth of neurites from primary neurons in culture therefore represents a regeneration process rather than the *de novo* formation of neurites. As a compromise between the strengths and weaknesses of those model systems, we chose P19 cells for our high-content screens, as they can initiate the first formation of neurites in cell culture and as they can differentiate into neurons more rapidly compared to toti-potent embryonic stem cells [22]. Furthermore, the onset of morphological differentiation approximately 3 days after transfection of siRNA oligonucleotides forms a good compromise between efficient protein knockdown and variability due to prolonged culturing in screening conditions.

Based on our observations, the overall process of neurite formation can be divided into 2 functional layers: a) regulation of microtubule stability and b) motor generated forces which shift microtubules against the cell cortex. Several microtubule stabilizing proteins, which were previously suggested to play a role in neuromorphogenesis, such as doublecortin (*Dcx*) [24], MAP1B (*Mtap1b*) [25] and *Clasp* [26] were found to contribute to neurite outgrowth, as their depletion reduced average neurite length (Table S4). These observations suggest that one key requirement for neurite initiation and neurite outgrowth is regulation of microtubule stability. Our epistasis analysis suggests that this essential cellular process can be both positively and negatively modulated via competition between EB1 and EB2 for binding sites on microtubules.

Motor proteins play an important role not only in intracellular transport processes, but also in the spatial organization of cytoskeletal structures. A particularly well-studied example is the formation of the mitotic spindle, which emerges from interactions between dynamic microtubules and motor proteins via self-organization [27]. In our earlier studies, we proposed that cortical dynein complexes play a role in the initiation of neurites by pushing microtubule arrays with leading plus-ends into newly formed protrusions [4]. Other studies also highlight a role for cortical dynein in related processes, such as in growth cone turning or axon retraction [28], [29], microtubule-filopodia alignment [30] microtubule transport [31] or directed cell movement [32]. We now show that a specific set of cytoplasmic dynein subunits is required for neurite formation in differentiated P19 stem cells. The subunits that were found to be relevant for this process, including the heavy chain *Dync1h1*, the intermediate chain *Dync1i2* and the light intermediate chain *Dync1li1* also have more general functions in vesicular transport [19], [33]. Highly specialized dynein subunits, which drive retrograde intraflagellar transport, including *Dync2h1* and *Dync2li1* are indeed not required for neurite formation. Likewise, the intermediate chain 1, which functions in axonal signaling endosome transport [19], and the *Dctn5* subunit, which plays a specific role in nuclear movements in *Neurospora* [20] and in early and recycling endosome movements [21], also do not seem to play a role in the early stages of neurite outgrowth. This suggests that the core composition of cortical dynein complexes that can push microtubules to facilitate neurite formation is similar to the core composition of ubiquitously expressed dynein complexes. Additional factors that associate with those core subunits might add to complex specificity. Acto-myosin based contractility can produce an opposing force that counters this dynein mediated microtubule pushing mechanism [4], [28], [29]. Our observation that knockdown of the myosin activator *Rock1* increases neurite outgrowth is consistent with this idea. Thus, a tug-of-war between these opposing forces might play a role in the decision when and where a neurite is formed.

**Figure 5:**
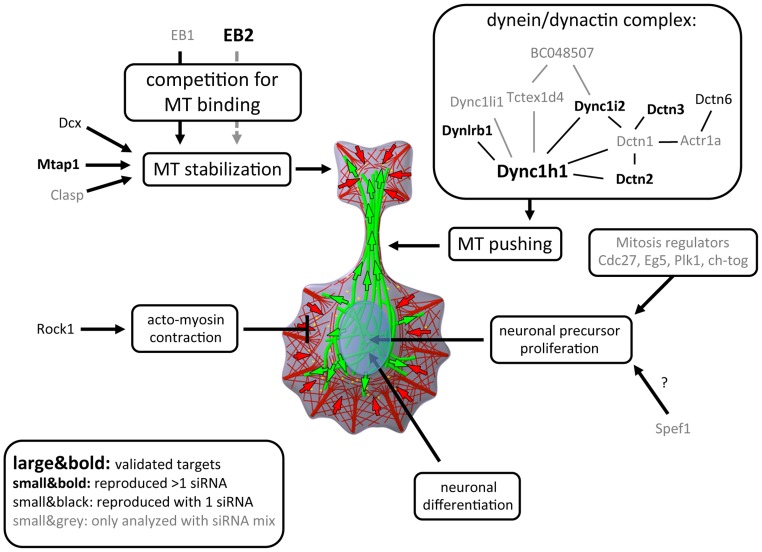
Proposed roles for cytoskeletal regulators in neurite formation. A model for the role of microtubules in neurite formation is presented. Several genes identified in this screen influence distinct functional layers of this process. The grey, dashed arrow pointing from EB2 indicates weak microtubule stabilizing activity compared to EB1. (Large&bold: gene targets, which were validated for multiple independent siRNAs via western blot analysis; small&bold: gene targets, which were reproduced with at least two independent siRNAs in secondary screens; small&black: gene targets, which were reproduced with one single siRNAs in secondary screens; small&grey: candidates, which were analyzed only with siRNA mixtures).

Based on this morphometric screen, we propose a model for the role of microtubule regulators in neurite formation (Figure 5). In the first layer of regulation, microtubules are stabilized to enable outgrowth of microtubule arrays that will later form the shaft of newly formed neurites. In this study, a potential mechanism to control microtubule stability was identified, by which EB isoforms with differing stabilizing activity compete for limited binding sites on microtubules. The observation, that knockdown of EB1 or EB2 can modulate neurite outgrowth either negatively or positively, underscores their central role for this first layer of microtubule regulation. Stabilized microtubules can grow more persistently towards the periphery, but contractile forces and retrograde flow generated by the actin cytoskeleton counteract this growth near the cell border. However, in the second layer of microtubule regulation, growing microtubules that point with their plus-ends towards the cell periphery are linked to the cell cortex and pushed further towards the periphery by cortical dynein complexes. Our study identified dynein complex subunits, which are required for this second layer of microtubule regulation. These layers of microtubule regulation act synergistically to initiate neurites by shifting stabilized microtubules towards the cell periphery to overcome inhibitory actin-based contractile forces.

## Materials and Methods

### Plasmid Construction

pUB-NeuroD2 was generated by inserting the *NeuroD2* coding sequence from pCS2-NeuroD2 [6] into pUB-GFP [34] via EcoRI and NotI restriction sites.

### Cell Culture and Transfection

P19 cells were obtained from ATCC (Teddington, UK) and cultured in growth medium (MEM Eagle + glutamate/pyruvate, 10% FBS and penicillin/streptomycin; all media components were obtained from Pan Biotech, Aidenbach, Germany). For reverse co-transfection of P19 cells with neurogenic transcription factor NeuroD2 and siRNA oligonucleotides, indicated amounts of on-target Plus siRNA oligos (Dharmacon, Thermo Fischer, Lafayette, USA) were diluted into 5 µl clear MEM (PAN Biotech) and transferred into flat bottom 384-well plates (Art.-Nr.: 781092; Greiner, Frickenhausen, Germany) using an automated pipetting robot (MICROLAB(R)STAR line, Hamilton, Martinsried, Germany). In primary screens, mixtures of 4 independent siRNA oligos were used (SMARTpools, Dharmacon) and in secondary screens, the individual siRNAs from these mixtures were applied in separate wells. siRNA sequences targeting Mapre2 were: oligo A: AGACAUUCGCUCACGGACA; oligo B: CCAGAGAAUUGGAACGUGU; oligo C: GUGUAUUCUACCUCGAUAA; oligo D: GCAAUUCAUCAACGGGAAA. siRNA sequences targeting Dync1h1 were: oligo A: GAAAUCAACUUGCCCGAUA; oligo B: UCACACACGUGCUGAGAAA; oligo C: CCAACCAGCUCUACCCAUU; oligo D: CAACAUAGACAUUCAUUAC. Plates were kept at –80°C and thawed immediately before use. To each well, 50 ng pUB-NeuroD2 and 10 ng pUB-GFP were added in 5 µl clear MEM followed by 120 nl Lipofectamine 2000 (Invitrogen, Karlsruhe, Germany) in 5 µl clear MEM (preincubated for 5 minutes according to manufacturer’s instructions). Plates were incubated for 20-30 minutes and 8000 undifferentiated P19 cells were plated per well in 85 µl serum-reduced differentiation medium (clear MEM + glutamate/pyruvate, 5% FBS, without penicillin/streptomycin). For confocal microscopy, reverse transfection was performed in 8-well Lab-Tek dishes (Thermo Scientific) using 24000 undifferentiated P19 cells in 400 µl medium, 480 ng pUB-NeuroD2, 16 pmol of siRNA pool, 20 µl clear MEM and 480 nl Lipofectamine 2000 per well.

### Immunocytochemistry

P19 cells were grown for 4 days and fixed using 4% formaldehyde/PBS at 37°C for 20 min. Cells were permeabilized using 0.25% Triton-X-100/PBS for 10 minutes, followed by blocking in 10% BSA/PBS and primary and secondary antibody incubation in 2% BSA/PBS. Mouse monoclonal anti-β-III-tubulin antibodies (TU-20, Exbio, Vestec, Czech Republic) were used at 1∶2000 dilution and Alexa 568-labeled goat anti-mouse antibodies (Invitrogen) were used as secondaries at 1∶1000 dilution. For autofocusing, cells were counterstained with Hoechst 33258 (Sigma Aldrich, Steinheim, Germany). For analysis of siRNA knockdown selectivity, cells were stained both with mouse anti-β-III-tubulin [1∶2000] and rabbit anti-MAP2 antibodies ([1∶2000]; antiserum 266, kind gift of Shelley Halpain) in combination with Alexa 568-labeled goat anti-mouse and Alexa 750-labeled goat anti-rabbit antibodies (Invitrogen, both at 1∶1000 dilution). For confocal microscopic analysis of EB1 or EB2 levels, cells cultured on Lab-Tek dishes were stained with rat anti-EB1 ([1∶500] clone KT51, Absea, Beijing, China) or rat anti-EB2 antibodies ([1∶500] clone KT52, Absea, Beijing, China) in combination with mouse anti-β-III-tubulin antibodies [1∶2000]. Alexa 488-labeled goat anti-rat and Alexa 568-labeled goat anti-mouse antibodies (Invitrogen, both at 1∶1000 dilution) were used as secondaries. To preserve subcellular localization of EB proteins and to partially extract the cytosolic fraction, fixation was performed using 100% methanol for 10min at –20°C. As indicated, in some experiments fixation was also performed using 4% formaldehyde/PBS at 37°C for 20 min to preserve overall protein expression levels.

### Microscopy

Automated microscopy was performed on an IX81 microscope (Olympus, Hamburg) equipped with a 10x PlanApo Objective (UPlans APO 10x NA 0.4) and an Orca ER C4742-80-12AG camera (Hamamatsu, Herrsching am Ammersee, Germany). Automated scanning of 384-well plates was done via the ScanR software (Olympus). To ensure optimal spectral separation, images were obtained with sets of excitation/emission filters and dichroic mirrors from Chroma (AHF Analysentechnik, Tübingen) optimized for each fluorophore. Confocal scanning microscopy was performed on a Zeiss LSM510 microscope equipped with 40x or 63x C-Apochromat Objectives (NA 1.2). The 488 nm line of a Argon laser, the 561 nm and 405 nm lines from DPSS 561-10 and K05-30 diode lasers were used to excite Alexa 488, Alexa 568 and Hoechst 33258 fluorophores respectively.

### Western Blotting

P19 cells were differentiated by co-transfection of 4 µg pUB-NeuroD2 and 160 pmol siRNA oligonucleotides in 6-well dishes and incubation for 4 days at 37°C. Lysates were prepared by solubilization in boiling hot 10% SDS/50 mM Tris-HCl (detection of DYNC1H1 and GAPDH) or ice-cold RIPA buffer (detection of EB2 and GAPDH), dissolved in SDS-sample buffer and resolved in ready-to-use 4–15% gradient gels (BioRad, München, Germany). Subsequently proteins were transferred onto polyvinylidine Fluoride (PVDF) membranes (Millipore, Schwalbach, Germany) at 100 V for 1h. After blocking (Odyssey(R) blocking buffer, LI-COR, Lincoln, USA), membranes were incubated with primary antibodies against DYNC1H1 (Dynein heavy chain rabbit polyclonal antibody R-325 at 1∶200, Santa Cruz Biotechnology), EB2 (EB2 rat polyclonal antibody KT-52 at 1∶2000, Absea, Beijing, China), EB1 (EB1 rat polyclonal antibody KT-51 at 1∶3000, Absea, Beijing, China) and GAPDH (GAPDH mouse monoclonal antibody at 1∶1000, Pierce-Antibody, Thermo Scientific, Rockford, USA or GAPDH 14C10 rabbit polyclonal antibody at 1∶1000, Cell Signaling Technology, Danvers, USA). Odyssey IRDye680 goat anti mouse, IRDye680 goat anti rat or IRDye800 goat anti mouse/rabbit (LI-COR, Lincoln, USA) were used as secondary antibodies. Immunoreactivity was recorded using the LI-COR infrared scanner (LI-COR). In some experiments, peroxidase-linked secondary antibodies were used (Peroxidase-conjugated AffiniPure Goat anti-Mouse IgG + IgM (H+L) at 1∶1000, Jackson ImmunoResearch, USA or goat anti-rat IgG, HRP conjugate at 1∶5000, Millipore, USA) and luminescent signals were detected via Pierce ECL Western Blotting Substrate (Thermo Scientific). Band intensities were quantified using the ImageJ Software. All measurements were normalized to GAPDH levels.

### Data Analysis

The total neurite length (in pixels), total neuronal cell body area (in pixels) and total fluorescence signal (counts) per microscopic field were measured using the NeuriteQuant package [7]. In addition, the total fluorescence intensity derived from the neuronal marker TU-20 and the total fluorescence intensity derived from co-transfected EGFP-coding plasmids were determined per field after subtraction of average background levels. Morphometric and intensity measurements were averaged on a per well basis by calculating the mean values from 6 microscopic fields. In addition, mean values and standard deviations were calculated on a per plate basis from 20 positive control wells (no siRNA, but with NeuroD2) and 20 negative control wells (neither siRNA nor NeuroD2). All morphometric and intensity measurements were transformed into percent on the basis of averages from positive and negative controls.

An estimate of overall growth of transfected cells was determined as the fluorescence intensity of co-transfected EGFP (in %). For each well, the difference of that measurement from the mean value of positive controls was determined. Neuronal differentiation was determined by measuring the relative change in the total neuronal cell body area (in %) vs. the background-corrected EGFP fluorescence intensity (in %), each on a per (microscopic) field basis. Analogously, neurite outgrowth was determined by measuring the relative change in the total neurite length (in %) vs. the total neuronal cell body area (in %), also on a per field basis. To sensitively detect relative changes in these measurements in comparison to control measurements, a linear regression of the 20 positive control values from each plate was first determined. For corresponding measurements from each well in that plate, the deviation from this regression line was calculated as d = (m*x-y+b)/sqrt(m^2^+1) with m, b: slope and y-intercept of the regression line; x, y: background-corrected EGFP fluorescence intensity and total neuronal cell body area (determination of neuronal differentiation) or total neuronal cell body area and total neurite length (determination of neurite outgrowth). The deviations of morphometric measurements from positive controls, which are measured by these distances, are visually represented in Figure 2 B and C as grey arrows, respectively.

All measurements were divided by the standard deviation of positive controls to express them in units of standard deviations. A secondary screen was performed to test, if phenotypes obtained in the primary screen using mixtures of 4 siRNA oligonucleotides can be reproduced using individual siRNAs. The criterion for phenotype classification was modulation of the respective measurements by more than 3 standard deviations compared to positive controls. In this secondary screen the majority of phenotypes (∼73%; 22 out of 30) was reproduced at least by one siRNA oligonucleotide (Tables S6 and S7). For ∼36% of target genes the phenotype was reproduced with multiple oligonucleotides (11 out of 30). Only in two cases, opposite phenotypes were observed for individual siRNAs – however also in these cases the overall trend corresponded to the expectation from the primary screen: 2 siRNAs targeting Keg1 induced expected elongation of neurites, while one siRNA induced shorter neurites. 3 siRNAs targeting *Tpx2* induced the expected shortening of neurites, while 1 siRNA induced longer neurites. The primary and secondary screens were repeated 3 times and mean values and standard error of the mean are indicated for each measurement of each condition. For genetic epistasis analyses, siRNA oligonucleotides targeting one or more genes were combined and supplemented with non-targeting siRNA oligonucleotides to a total amount of 4pmol and processed and analyzed analogously to the primary and secondary screens. Statistical analyses of morphological measurements were performed via one-way ANOVA using the software Prism (GraphPad, La Jolla, USA).

As indicated, in some experiments average fluorescence intensity within neurites was determined by a modified NeuriteQuant macro that produces a mask by thresholding and binarizing the neuronal β-III-tubulin image, applying this mask to the fluorescence image of interest (i.e. EB1 or EB2) and measuring the average fluorescence intensity within this masked region.

## Supporting Information

Figure S1 **Expression of differentiation related markers in P19 cells following transfection with NeuroD2.** Top: Microscopic image of P19 cells, which were cultured for 4 days after transfection with the neurogenic transcription factor NeuroD2. Bottom: Enlarged view of the frame area from top panels. Cyan arrows: OCT4 negative, TUBB3 positive neuronal cell. Yellow arrows: OCT4 positive, TUBB3 negative stem cell.
(JPG)

Figure S2 **Selective protein depletion during P19 cell differentiation.** Microscopic images of P19 cells, which were co-transfected with the neurogenic transcription factor NeuroD2 and either non-targeting or siRNA mixtures targeting the microtubule associated protein 2 (*Mtap2*) or neuronal β-III-tubulin (*Tubb3*). siRNA oligonucleotide mixtures (4pmol/well) efficiently and selectively depleted their corresponding target protein.
(JPG)

Figure S3 **Quantification of EB1 and EB2 protein depletion.** Lysates of neuronal differentiated P19 cells treated with siRNAs targeting EB1 or EB2 were analyzed via western blot analysis. Controls were either treated with non-targeting or no siRNA. A: Images of representative blots probed with anti-EB1 or anti-EB2. Anti-GAPDH was used as a loading control. B: Graphs showing average signals from 3 independent knockdown experiments normalized to GAPDH amounts.
(JPG)

Figure S4 **Immunocytochemical analysis of EB1 and EB2 levels after EB2 protein depletion in formaldehyde fixed cells.** A: Confocal z-projections of neuronal differentiated P19 cells stained with antibodies for neuronal β-III-tubulin (Tubb3) and EB1 or EB2. Note that localization of EB proteins to microtubule plus tips is not preserved in formaldehyde fixed samples. Therefore, immunoreactivity represents overall protein levels and not subcellular localization. See Figure 3D and Figure 3E for analysis of partially extracted, methanol fixed samples in which microtubule plus-tip binding is preserved. B: Quantification of average EB1 and EB2 signals within neurites of P19 cells. Confocal z-projections of EB1 or EB2 signals were masked based on the neuronal β-III-tubulin signal and average intensities were calculated within those masked regions (***: p<0.001; Student’s t-test, data obtained from 3 independent knock-down experiments).
(JPG)

Table S1 Microtubule-related genes which are required for proliferation of precursors. Shown is the decrease in proliferation efficiency in standard deviations±standard error of 3 repetitions. Only candidates that deviate from controls by more than 3 standard deviations on average are shown. Stringent candidates (SD-SEM>3) are bold and marginal candidates (SD-SEM<3) are in regular font.
(DOC)

Table S2 Microtubule-related genes, which positively modulate neuronal differentiation. Shown is the decrease in neuronal differentiation in standard deviations±standard error of 3 repetitions. Genes, that on average show a more than 3 standard deviation reduction in proliferation efficiency (see Table S1), were excluded from this analysis as the strong reduction of measurable cells associated with inhibition of precursor growth prevents reliable quantitative analysis of neuronal differentiation efficiency.
(DOC)

Table S3 Microtubule-related genes, which negatively modulate neuronal differentiation. Shown is the increase in neuronal differentiation in standard deviations±standard error of 3 repetitions.
(DOC)

Table S4 Microtubule-related genes, which positively modulate neurite outgrowth. Shown is the decrease in average neurite length (distance from regression line in standard deviations±standard error of 3 repetitions). Only candidates that deviate from controls by more than 3 standard deviations on average are shown. Stringent candidates (SD-SEM>3) are bold and marginal candidates (SD-SEM<3) are in regular font. Genes, that on average show a more than 3 standard deviation reduction in proliferation efficiency (see Table S1), were excluded from this analysis as the strong reduction of measurable cells associated with inhibition of precursor growth prevents reliable quantitative analysis of neurite outgrowth.
(DOC)

Table S5 Microtubule-related genes, which negatively modulate neurite outgrowth. Shown is the increase in average neurite length (distance from regression line in standard deviations±standard error of 3 repetitions). Only candidates which deviate from controls by more than 3 standard deviations on average are shown. Stringent candidates (SD-SEM>3) are bold and marginal candidates (SD-SEM<3) are in regular font.
(DOC)

Table S6 Single oligo knockdown of microtubule-related genes, which negatively modulate neurite outgrowth. Selected candidate genes, identified in the primary screen, for which siRNA-mediated knockdown lead to an increase in the average neurite length, were targeted with individual siRNAs. The increase in average neurite length (distance from regression line in standard deviations±standard error of 3 repetitions) is shown. Reproduced phenotypes are shown in bold black, opposite phenotpyes are shown in bold red.
(DOC)

Table S7 Single oligo knockdown of microtubule-related genes, which positively modulate neurite outgrowth. Selected candidate genes, identified in the primary screen, for which siRNA-mediated knockdown lead to a decrease in the average neurite length, were targeted with individual siRNAs. The decrease in average neurite length (distance from regression line in standard deviations±standard error of 3 repetitions) is shown. Reproduced phenotypes are shown in bold black, opposite phenotpyes are shown in bold red.
(DOC)
