# Supplementary figures and images for: A Morphometric Screen Identifies Specific Roles for Microtubule-Regulating Genes in Neuronal Development of P19 Stem Cells
Source: PLoS One. 2013 Nov 18;8(11):e79796. doi: 10.1371/journal.pone.0079796 (PMC3832585; doi:10.1371/journal.pone.0079796)

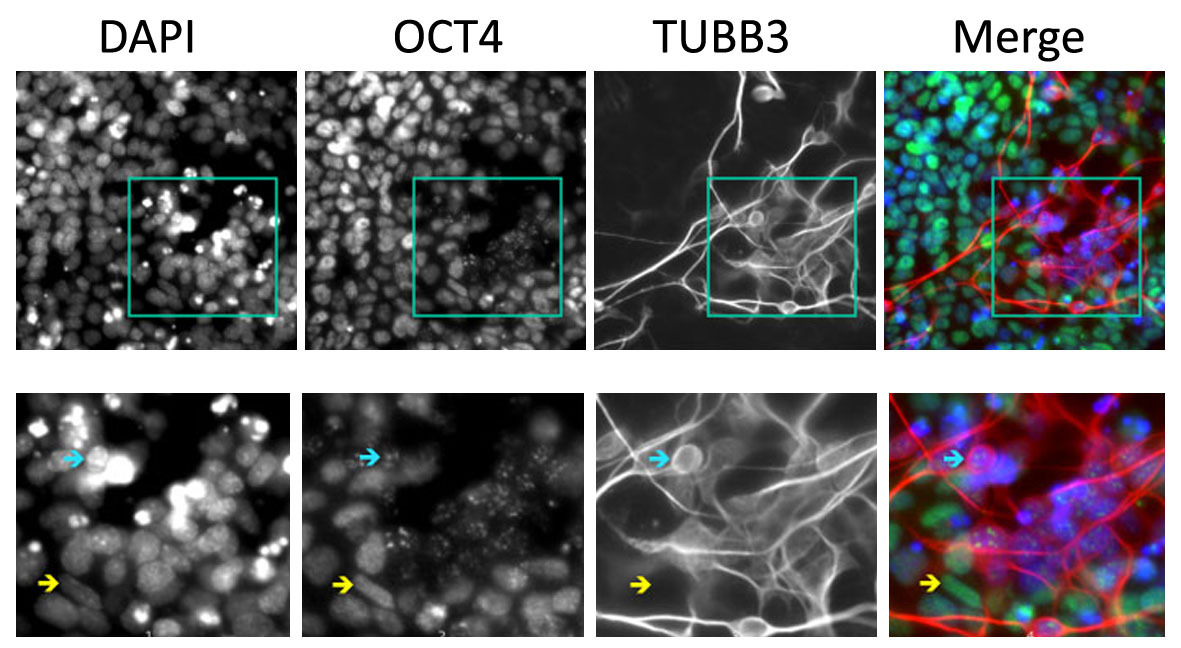

Supplement: Figure S1 — Expression of differentiation related markers in P19 cells following transfection with NeuroD2. Top: Microscopic image of P19 cells, which were cultured for 4 days after transfection with the neurogenic transcription factor NeuroD2. Bottom: Enlarged view of the frame area from top panels. Cyan arrows: OCT4 negative, TUBB3 positive neuronal cell. Yellow arrows: OCT4 positive, TUBB3 negative stem cell. (JPG) [file pone.0079796.s001.jpg]

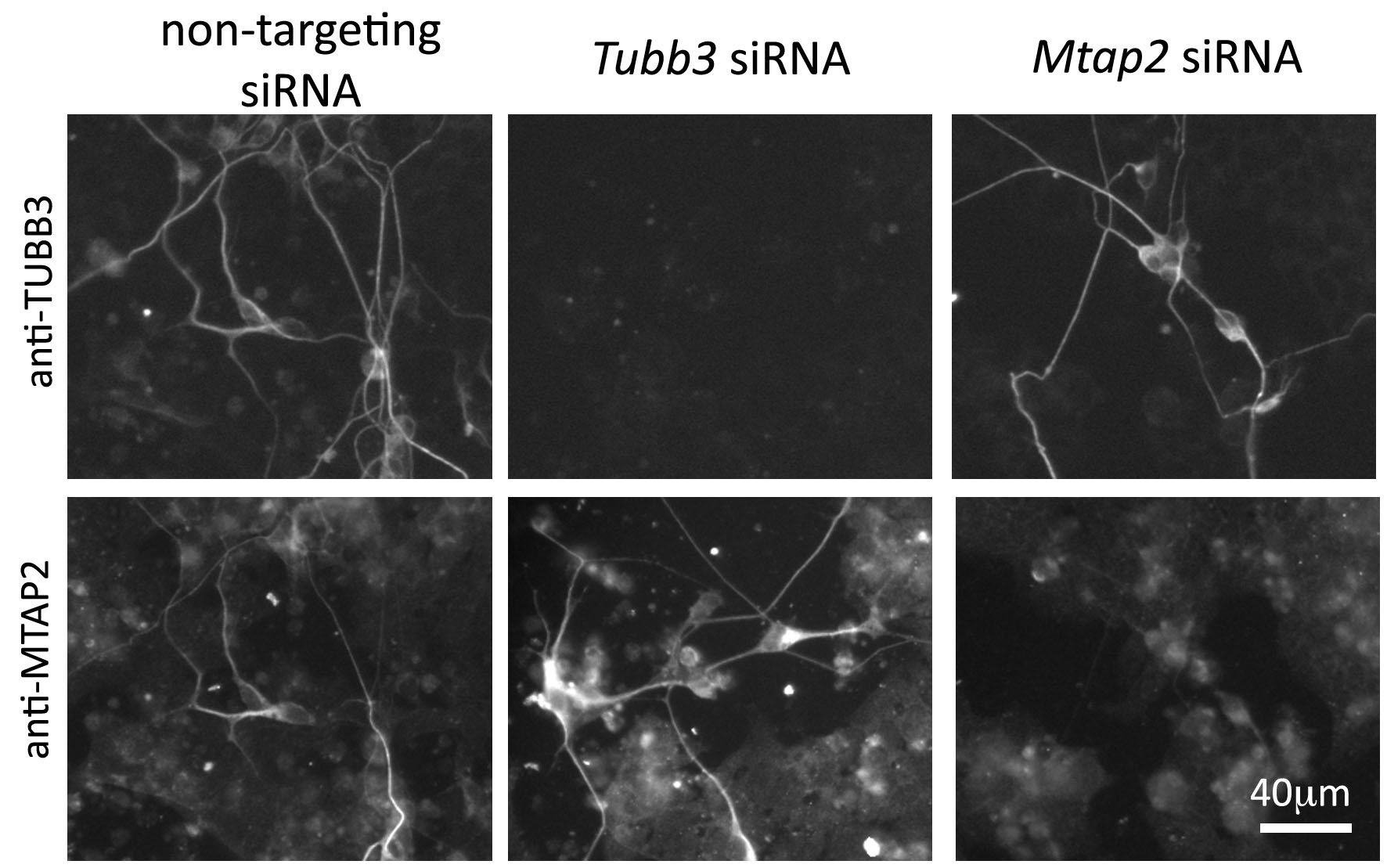

Supplement: Figure S2 — Selective protein depletion during P19 cell differentiation. Microscopic images of P19 cells, which were co-transfected with the neurogenic transcription factor NeuroD2 and either non-targeting or siRNA mixtures targeting the microtubule associated protein 2 (Mtap2) or neuronal β-III-tubulin (Tubb3). siRNA oligonucleotide mixtures (4pmol/well) efficiently and selectively depleted their corresponding target protein. (JPG) [file pone.0079796.s002.jpg]

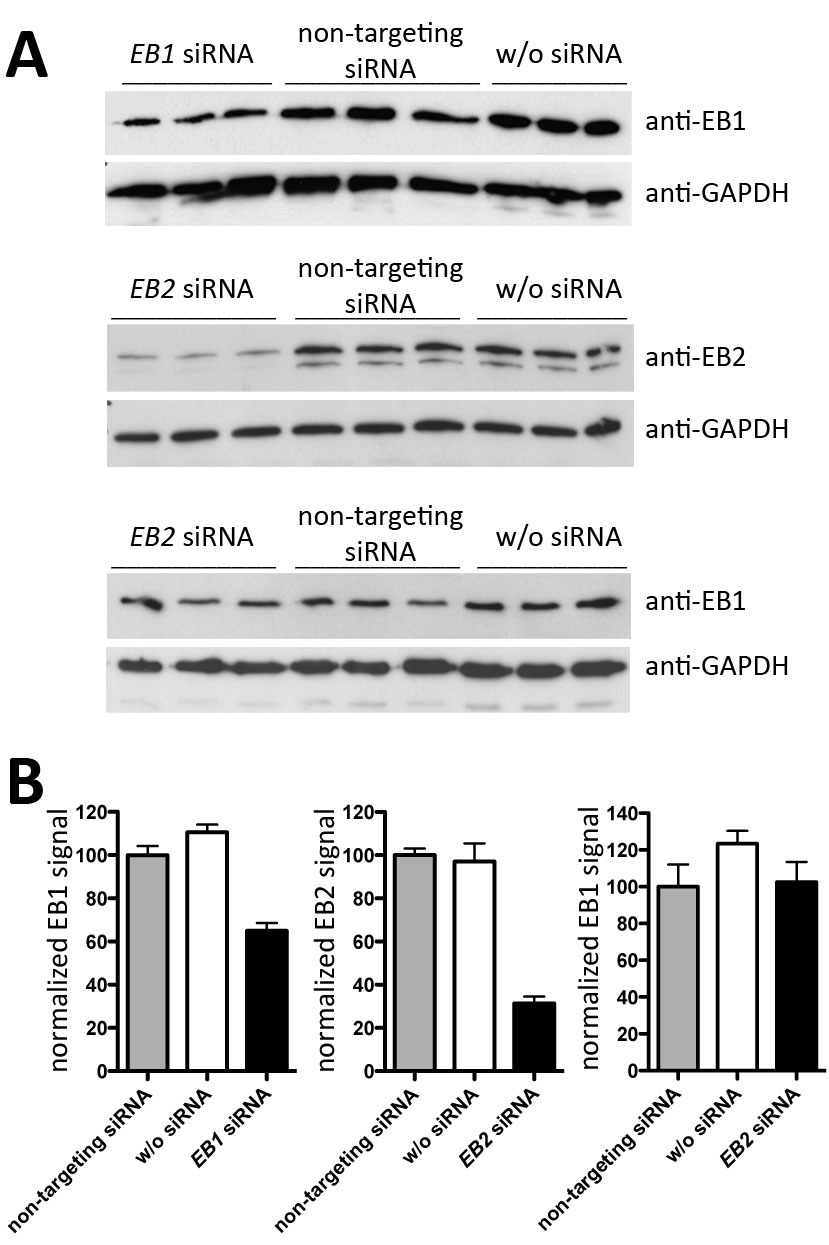

Supplement: Figure S3 — Quantification of EB1 and EB2 protein depletion. Lysates of neuronal differentiated P19 cells treated with siRNAs targeting EB1 or EB2 were analyzed via western blot analysis. Controls were either treated with non-targeting or no siRNA. A: Images of representative blots probed with anti-EB1 or anti-EB2. Anti-GAPDH was used as a loading control. B: Graphs showing average signals from 3 independent knockdown experiments normalized to GAPDH amounts. (JPG) [file pone.0079796.s003.jpg]

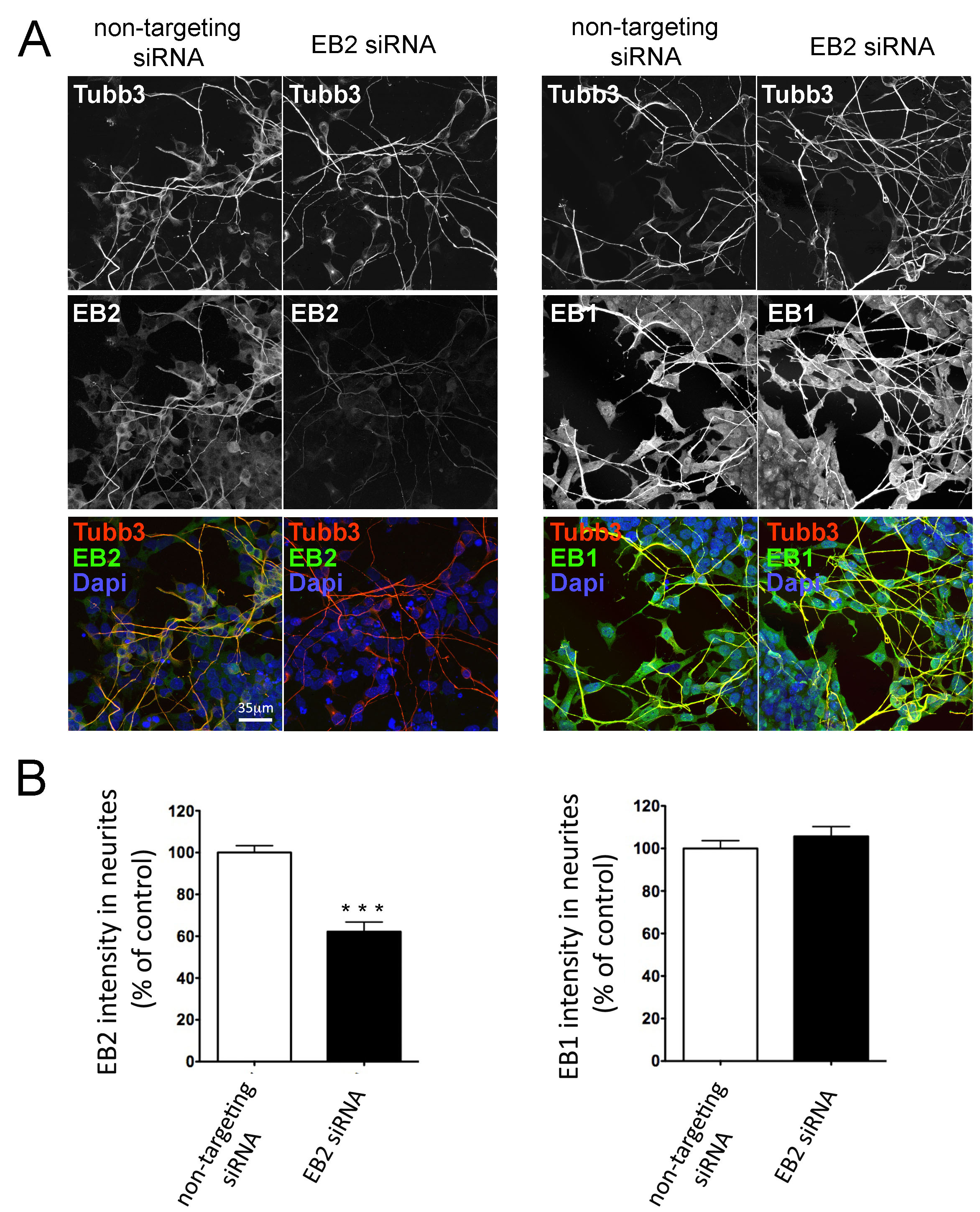

Supplement: Figure S4 — Immunocytochemical analysis of EB1 and EB2 levels after EB2 protein depletion in formaldehyde fixed cells. A: Confocal z-projections of neuronal differentiated P19 cells stained with antibodies for neuronal β-III-tubulin (Tubb3) and EB1 or EB2. Note that localization of EB proteins to microtubule plus tips is not preserved in formaldehyde fixed samples. Therefore, immunoreactivity represents overall protein levels and not subcellular localization. See Figure 3D and Figure 3E for analysis of partially extracted, methanol fixed samples in which microtubule plus-tip binding is preserved. B: Quantification of average EB1 and EB2 signals within neurites of P19 cells. Confocal z-projections of EB1 or EB2 signals were masked based on the neuronal β-III-tubulin signal and average intensities were calculated within those masked regions (***: p<0.001; Student’s t-test, data obtained from 3 independent knock-down experiments). (JPG) [file pone.0079796.s004.jpg]
