# Supplementary material for: A Morphometric Screen Identifies Specific Roles for Microtubule-Regulating Genes in Neuronal Development of P19 Stem Cells
Source: PLoS One. 2013 Nov 18;8(11):e79796. doi: 10.1371/journal.pone.0079796 (PMC3832585; doi:10.1371/journal.pone.0079796)
Supplement: Table S1 — Microtubule-related genes which are required for proliferation of precursors. Shown is the decrease in proliferation efficiency in standard deviations±standard error of 3 repetitions. Only candidates that deviate from controls by more than 3 standard deviations on average are shown. Stringent candidates (SD-SEM>3) are bold and marginal candidates (SD-SEM<3) are in regular font. (DOC) [file pone.0079796.s005.doc]

| Gene Symbol | Reduction [SD±SEM] | Description of Gene |
| --- | --- | --- |
| ***Kif11*** | **5.68±0.55** | **Eg5 mitotic kinesin** |
| ***Kif11* (@ 2pmol)** | **4.96±0.40** | **Eg5 mitotic kinesin** |
| ***Cdc27*** | **4.67±0.51** | **APC3 – essential cell cycle regulator** |
| ***Spef1*** | **3.86±0.43** | **sperm flagellar 1** |
| *Dync1i1* | 3.53±0.76 | dynein subunit, intermediate chain 1 |
| *Dync1i2* | 3.53±0.76 | dynein subunit, intermediate chain 2 |
| *Plk1* | 3.47±1.03 | polo-like kinase 1 |
| *Ckap5* | 3.34±0.71 | ch-TOG, mammalian XMAP215 homolog |
| *Neil2* | 3.34±0.71 | Also known as GM1212 |
| *Ubn2* | 3.20±0.66 | ubinuclein, histone chaperones |
| *Vps41* | 3.15±0.32 | Vps41, subunit of the HOPS complex |
| *Gabarap* | 3.03±0.52 | regulator of autophagy |
| *Mtap2* | 3.03±0.52 | Map2, stabilizes/nucleates MTs |
