# Supplementary material for: A Morphometric Screen Identifies Specific Roles for Microtubule-Regulating Genes in Neuronal Development of P19 Stem Cells
Source: PLoS One. 2013 Nov 18;8(11):e79796. doi: 10.1371/journal.pone.0079796 (PMC3832585; doi:10.1371/journal.pone.0079796)
Supplement: Table S2 — Microtubule-related genes, which positively modulate neuronal differentiation. Shown is the decrease in neuronal differentiation in standard deviations±standard error of 3 repetitions. Genes, that on average show a more than 3 standard deviation reduction in proliferation efficiency (see Table S1), were excluded from this analysis as the strong reduction of measurable cells associated with inhibition of precursor growth prevents reliable quantitative analysis of neuronal differentiation efficiency. (DOC) [file pone.0079796.s006.doc]

| Gene Symbol | Reduction [SD±SEM] | Description of Gene |
| --- | --- | --- |
| *Bcl2l11* | 3.10±0.28 | Pro-apoptotic mediator Bim |
| *Mtap1b* | 2.49±0.36 | Microtubule associated protein 1B |
| *Kif3c* | 2.34±0.82 | Kinesin family member 3C |
