# Supplementary material for: A Morphometric Screen Identifies Specific Roles for Microtubule-Regulating Genes in Neuronal Development of P19 Stem Cells
Source: PLoS One. 2013 Nov 18;8(11):e79796. doi: 10.1371/journal.pone.0079796 (PMC3832585; doi:10.1371/journal.pone.0079796)
Supplement: Table S3 — Microtubule-related genes, which negatively modulate neuronal differentiation. Shown is the increase in neuronal differentiation in standard deviations±standard error of 3 repetitions. (DOC) [file pone.0079796.s007.doc]

| Gene Symbol | Increase [SD±SEM] | Description of Gene |
| --- | --- | --- |
| *Gabarap* | 3.89±1.08 | regulator of autophagy |
| *Tubgcp4* | 3.61±1.50 | Tubulin ring complex component |
| *Gabarap* (@ 2pmol) | 3.17±1.09 | Regulator of autophagy |
| *Tubb2b* | 3.09±0.43 | Tubulin, beta 2B |
| *Macf1* | 3.07±1.25 | Microtubule actin crosslinking factor 1 |
| *Tubb5* | 2.39±0.91 | Tubulin, beta 5 |
