# Supplementary material for: A Morphometric Screen Identifies Specific Roles for Microtubule-Regulating Genes in Neuronal Development of P19 Stem Cells
Source: PLoS One. 2013 Nov 18;8(11):e79796. doi: 10.1371/journal.pone.0079796 (PMC3832585; doi:10.1371/journal.pone.0079796)
Supplement: Table S4 — Microtubule-related genes, which positively modulate neurite outgrowth. Shown is the decrease in average neurite length (distance from regression line in standard deviations±standard error of 3 repetitions). Only candidates that deviate from controls by more than 3 standard deviations on average are shown. Stringent candidates (SD-SEM>3) are bold and marginal candidates (SD-SEM<3) are in regular font. Genes, that on average show a more than 3 standard deviation reduction in proliferation efficiency (see Table S1), were excluded from this analysis as the strong reduction of measurable cells associated with inhibition of precursor growth prevents reliable quantitative analysis of neurite outgrowth. (DOC) [file pone.0079796.s008.doc]

| Gene Symbol | Reduction [SD±SEM] | Description of Gene |
| --- | --- | --- |
| ***Dctn3*** | **7.93±2.19** | **dynactin 3, p24/22** |
| ***Dctn2*** | **6.93±2.43** | **dynactin 2, dynamitin, p50** |
| ***Incenp*** | **6.01±1.27** | **inner centromere protein** |
| *Nefl* (@1pmol) | 5.41±3.19 | neurofilament, light polypeptide |
| ***Incenp* (@2pmol)** | **5.20±1.88** | **inner centromere protein** |
| ***Dync1h1*** | **5.20±0.87** | **dynein cytoplasmic 1 heavy chain 1** |
| ***Kif3c*** | **5.12±0.97** | **kinesin family member 3C** |
| ***Dctn4*** | **5.08±0.99** | **dynactin 4, p62** |
| ***Ctnnd1*** | **5.04±2.03** | **catenin (cadherin associated protein), delta 1** |
| ***Poc5*** | **4.93±1.52** | **centriolar protein homolog** |
| ***Cep170*** | **4.92±1.72** | **centrosomal protein 170** |
| ***Plk1*(@2pmol)** | **4.92±1.64** | **Polo-like kinase 1** |
| ***Macf1*** | **4.63±0.87** | **microtubule-actin crosslinking factor 1, Acf7** |
| *Dync1li1* | 4.62±1.87 | Dynein subunit, light intermediate chain 1 |
| *Kif23* | 4.51±1.25 | kinesin family member 23 (MKLP1) |
| ***Ncald* (@2pmol)** | **4.50±0.95** | **neurocalcin delta** |
| *Iigp1* | 4.49±1.71 | interferon inducible GTPase 1 |
| *Tekt4* (@1pmol) | 4.43±2.92 | tektin 4 |
| *Dctn3* (@1pmol) | 4.40±2.82 | dynactin 3, p24/22 |
| *Dcx* | 4.29±1.76 | doublecortin |
| ***Dctn6*** | **4.23±1.23** | **dynactin 6, p27** |
| ***Tcp1*** | **4.22±0.79** | **t-complex protein 1** |
| ***Tpx2*** | **4.18±1.06** | **microtubule-associated protein homolog** |
| *Mtap9* | 4.16±1.75 | microtubule-associated protein 9 |
| *Fam110c* | 4.08±1.92 | family with sequence similarity 110, member C |
| *Kifc2* | 4.01±1.20 | kinesin family member C2 |
| *Ctnnd1*(@2pmol) | 3.99±2.29 | catenin (cadherin associated protein), delta 1 |
| *Lrrc49* | 3.98±2.08 | leucine rich repeat containing 49 |
| *Cep57* (@2pmol) | 3.97±3.14 | centrosomal protein 57 |
| ***Ogg1* (@1pmol)** | **3.92±0.39** | **8-oxoguanine DNA-glycosylase 1** |
| *Tctex1d4* | 3.89±1.18 | Tctex1 domain containing 4 |
| *Kif23* (@2pmol) | 3.83±1.95 | kinesin family member 23 (MKLP1) |
| ***Dynlt1b*** | **3.81±0.41** | **dynein light chain Tctex-type 1D** |
| ***Gabarap* (@1pmol)** | **3.79±0.18** | **regulator of autophagy** |
| *Tubgcp4* | 3.77±3.83 | Tubulin ring complex component |
| *Tpx2* (@0.5pmol) | 3.71±1.44 | microtubule-associated protein homolog |
| ***Mtap1b*** | **3.70±0.50** | **microtubule-associated protein 1B** |
| ***Dynlrb1*** | **3.69±0.39** | **dynein light chain roadblock-type 1** |
| *Clasp1* | 3.66±0.77 | CLIP associating protein 1 |
| *Mapre1* | 3.64±0.83 | EB family, member 1 |
| *Kif11* (@1pmol) | 3.52±3.09 | Eg5 mitotic kinesin |
| ***E230025N22Rik*** | **3.45±0.23** | **Riken cDNA E230025N22 gene** |
| ***Dync1i2* (@2pmol)** | **3.43±0.15** | **dynein cytoplasmic 1 intermediate chain 2** |
| *Tbccd1* (@1pmol) | 3.42±0.85 | TBCC domain containing 1 |
| *Dtnb* (@2pmol) | 3.39±1.65 | dystrobrevin, beta |
| *Tubb2b* | 3.39±1.32 | tubulin, beta 2B |
| *Rsn* (@0.5pmol) | 3.37±0.90 | restin, Clip1 |
| *Gabarap* (@0.5pmol) | 3.35±0.70 | regulator of autophagy |
| *Tbccd1* | 3.32±0.66 | TBCC domain containing 1 |
| *Crocc* | 3.32±0.87 | ciliary rootlet coiled-coil, rootletin |
| *Nde1* | 3.28±0.64 | nuclear distribution gene E homolog 1 |
| *Bcl2l11* | 3.26±1.57 | BCL2-like 11 (apoptosis facilitator) |
| *BC048507* | 3.25±1.51 | related to dynein light chain type 1 |
| *Eml6* | 3.25±2.30 | echinoderm microtubule associated protein like 6 |
| *Poc5* (@1pmol) | 3.24±0.57 | centriolar protein homolog |
| *Dnahc11* (@1pmol) | 3.23±0.35 | dynein, axonemal, heavy chain 11 |
| *Tekt4* | 3.21±2.01 | tektin 4 |
| *Cep57* | 3.20±1.11 | centrosomal protein 57 |
| *Eml4* | 3.17±0.50 | echinoderm microtubule associated protein like 4 |
| *Mark4* | 3.14±1.12 | MAP/microtubule affinity-regulating kinase 4 |
| *Apc* (@2pmol) | 3.14±3.63 | adenomatosis polyposis coli |
| *Actr1a* | 3.14±0.90 | ARP1 actin-related protein 1 homolog A |
| *Kifap3* | 3.11±1.18 | kinesin-associated protein 3 |
| *Atg4c* (@2pmol) | 3.09±2.73 | autophagy-related 4C |
| *Dnahc5* (@0.5pmol) | 3.09±1.22 | dynein, axonemal, heavy chain 5 |
| *Dnahc5* (@2pmol) | 3.08±0.84 | dynein, axonemal, heavy chain 5 |
| *Nefm* | 3.07±0.90 | neurofilament, medium polypeptide |
| *Dync1h1* (@2pmol) | 3.04±0.94 | dynein cytoplasmic 1 heavy chain 1 |
| *Kif1a* | 3.03±2.78 | kinesin family member 1A |
| *Apg4c* | 3.03±1.80 | autophagy-related 4C (yeast) |
| *Mtap7d1* | 3.02±0.61 | contains microtubule-associated protein domain |
| *Hap1* (@0.5pmol) | 3.01±0.74 | huntingtin-associated protein 1 |
| *Tubb5* | 3.00±0.95 | Tubulin, beta 5 |
