# Supplementary material for: A Morphometric Screen Identifies Specific Roles for Microtubule-Regulating Genes in Neuronal Development of P19 Stem Cells
Source: PLoS One. 2013 Nov 18;8(11):e79796. doi: 10.1371/journal.pone.0079796 (PMC3832585; doi:10.1371/journal.pone.0079796)
Supplement: Table S5 — Microtubule-related genes, which negatively modulate neurite outgrowth. Shown is the increase in average neurite length (distance from regression line in standard deviations±standard error of 3 repetitions). Only candidates which deviate from controls by more than 3 standard deviations on average are shown. Stringent candidates (SD-SEM>3) are bold and marginal candidates (SD-SEM<3) are in regular font. (DOC) [file pone.0079796.s009.doc]

| Gene Symbol | Increase[SD±SEM] | Description of Gene |
| --- | --- | --- |
| ***Tbata*** | **6.52±2.38** | **thymus, brain and testes associated (spatial)** |
| ***Mapre2*** | **5.27±0.82** | **EB family, member 2** |
| ***Ttll3*** | **4.53±1.49** | **tubulin tyrosine ligase-like family, member 3** |
| ***Keg1*** | **4.05±0.28** | **glycine N-acyltransferase-like protein** |
| *Dst* | 3.86±2.07 | dystonin, cytoskeletal crosslinker |
| *Serpina3c* (@2pmol) | 3.70±1.26 | serine (or cysteine) peptidase inhibitor |
| *Rock1* (@2pmol) | 3.59±0.79 | Rho kinase 1 |
| *Tubb2a* (@1pmol) | 3.46±1.02 | tubulin, beta 2A |
| *Vps4b* | 3.35±0.40 | vacuolar protein sorting 4b |
| *Tubb2a* (@1pmol) | 3.26±1.04 | tubulin, beta 2A |
| *Vps4b* (@2pmol) | 3.25±0.76 | vacuolar protein sorting 4b |
| *Disc1* | 3.16±0.15 | disrupted in schizophrenia 1 |
