# Supplementary material for: A Morphometric Screen Identifies Specific Roles for Microtubule-Regulating Genes in Neuronal Development of P19 Stem Cells
Source: PLoS One. 2013 Nov 18;8(11):e79796. doi: 10.1371/journal.pone.0079796 (PMC3832585; doi:10.1371/journal.pone.0079796)
Supplement: Table S6 — Single oligo knockdown of microtubule-related genes, which negatively modulate neurite outgrowth. Selected candidate genes, identified in the primary screen, for which siRNA-mediated knockdown lead to an increase in the average neurite length, were targeted with individual siRNAs. The increase in average neurite length (distance from regression line in standard deviations±standard error of 3 repetitions) is shown. Reproduced phenotypes are shown in bold black, opposite phenotpyes are shown in bold red. (DOC) [file pone.0079796.s010.doc]

| Gene Symbol | Increase in average neurite length [SD] | | | | | |
| --- | --- | --- | --- | --- | --- | --- |
|  | siRNA mix | siRNA A | siRNA B | siRNA C | siRNA D | reproduced |
| *Tbata* | 6.52±2.38 | 1.98±1.44 | **3.66±1.42** | 1.00±1.3 | 0.42±1.18 | 1x |
| *Mapre2* | 5.27±0.82 | -0.38±0.83 | **7.43±1.44** | **6.94±0.83** | **4.03±0.34** | 3x |
| *Keg1* | 4.05±0.28 | -0.33±1.54 | **3.79±1.74** | **3.64±0.88** | **-6.86±3.38** | 2x |
| *Dst* | 3.86±2.07 | 0.16±0.75 | -0.49±0.81 | -0.53±0.71 | **6.29±2.24** | 1x |
| *Rock1* | 3.59±0.79^a^ | **5.65±0.52** | 0.61±0.94 | -0.61±1.07 | -0.80±0.22 | 1x |
| *Tubb2a* | 3.26±1.04^b^ | 1.93±1.94 | **8.27±0.21** | 1.25±1.30 | 0.64±1.16 | 1x |
| *Vps4b* | 3.35±0.40 | 1.24±1.82 | -0.63±1.32 | 0.56±2.30 | 2.14±0.87 | no |
| *Disc1* | 3.16±0.15 | **4.01±0.85** | 2.28±1.70 | -1.37±0.82 | 2.83±0.59 | 1x |

^a^(@2pmol)

^b^(@1pmol)
