# Supplementary material for: A Morphometric Screen Identifies Specific Roles for Microtubule-Regulating Genes in Neuronal Development of P19 Stem Cells
Source: PLoS One. 2013 Nov 18;8(11):e79796. doi: 10.1371/journal.pone.0079796 (PMC3832585; doi:10.1371/journal.pone.0079796)
Supplement: Table S7 — Single oligo knockdown of microtubule-related genes, which positively modulate neurite outgrowth. Selected candidate genes, identified in the primary screen, for which siRNA-mediated knockdown lead to a decrease in the average neurite length, were targeted with individual siRNAs. The decrease in average neurite length (distance from regression line in standard deviations±standard error of 3 repetitions) is shown. Reproduced phenotypes are shown in bold black, opposite phenotpyes are shown in bold red. (DOC) [file pone.0079796.s011.doc]

| Gene Symbol | Decrease in average neurite length [SD] | | | | | |
| --- | --- | --- | --- | --- | --- | --- |
|  | siRNA mix | siRNA A | siRNA B | siRNA C | siRNA D | reproduced |
| *Dctn3* | 7.93±2.19 | **5.72±1.85** | **5.88±0.66** | 0.48±0.82 | 1.79±0.83 | 2x |
| *Dctn2* | 6.93±2.43 | **7.67±0.80** | 1.47±0.86 | **7.01±0.36** | **3.07±1.11** | 3x |
| *Incenp* | 6.01±1.27 | **6.16±1.68** | -0.74±0.78 | 1.17±1.41 | **11.24±1.32** | 2x |
| *Incenp^a^* | 5.20±1.88 | 0.16±1.60 | 0.70±0.54 | 0.83±1.01 | **9.64±1.76** | 1x |
| *Dync1h1* | 5.20±0.87 | **6.46±1.36** | **6.13±1.04** | 0.00±1.17 | 2.60±0.29 | 2x |
| *Kif3c* | 5.12±0.97 | 2.23±1.34 | 0.51±0.80 | -1.10±0.64 | **4.55±0.97** | 1x |
| *Dctn4* | 5.08±0.99 | -1.71±1.65 | 0.96±1.55 | 0.67±0.42 | -0.75±0.56 | no |
| *Poc5* | 4.93±1.52 | -0.20±0.61 | 1.28±0.60 | -0.14±1.80 | 1.34±1.46 | no |
| *Macf1* | 4.63±0.87 | -0.70±0.70 | -0.66±2.21 | 0.13±0.59 | 0.93±0.44 | no |
| *Kif23* | 4.51±1.25 | **5.09±0.52** | **7.57±0.60** | -0.48±1.65 | 1.19±0.65 | 2x |
| *Ncald^a^* | 4.50±0.95 | -0.20±0.96 | -0.68±0.96 | 2.44±0.18 | -0.21±2.26 | no |
| *Dcx* | 4.29±1.76 | -0.83±0.85 | **3.58±1.10** | 1.95±0.76 | -0.87±1.55 | 1x |
| *Dctn6* | 4.23±1.23 | 0.69±0.64 | -2.08±0.45 | 2.43±0.06 | **3.17±0.93** | 1x |
| *Tpx2* | 4.18±1.06 | **4.66±0.42** | **7.54±1.10** | **6.39±0.84** | **-3.02±2.09** | 3x |
| *Dynlt1b* | 3.81±0.41 | -0.54±0.93 | -0.58±1.54 | -0.14±0.72 | 1.74±0.50 | no |
| *Gabarap^b^* | 3.79±0.18 | -0.17±0.79 | 0.66±0.14 | 0.38±0.79 | 0.60±1.10 | no |
| *Mtap1b* | 3.70±0.50 | 2.71±0.38 | **3.66±1.16** | 2.67±1.44 | **5.60±1.23** | 2x |
| *Dynlrb1* | 3.69±0.39 | **3.82±0.95** | **5.44±0.57** | 1.77±0.39 | 2.07±0.56 | 2x |
| *Mapre1* | 3.64±0.83 | -0.06±0.40 | 1.33±1.38 | **3.13±0.99** | 1.23±1.27 | 1x |
| *Dync1i2^a^* | 3.43±0.15 | 1.44±0.41 | 1.10±0.30 | **5.30±1.29** | **4.55±1.13** | 2x |
| *Gabarap^c^* | 3.35±0.70 | -1.81±0.62 | -0.83±1.12 | 0.89±1.75 | -1.67±1.18 | no |
| *Nde1* | 3.28±0.64 | -2.54±0.50 | 0.14±0.90 | -0.33±1.14 | **4.56±0.44** | 1x |
| *Dnahc11^b^* | 3.23±0.35 | -0.80±0.87 | 0.64±0.81 | 2.75±1.64 | 2.47±0.47 | no |
| *Eml4* | 3.17±0.50 | 2.11±1.00 | 2.23±1.71 | **4.84±0.57** | 1.91±0.79 | 1x |

^a^(@2pmol)

^b^(@1pmol)

^c^(@0.5pmol)
